# Spontaneous Pneumomediastinum with a Rare Presentation

**DOI:** 10.1155/2014/451407

**Published:** 2014-05-20

**Authors:** Ehsan Bolvardi, Elham Pishbin, Mohsen Ebrahimi, Azadeh Mahmoudi Gharaee, Farhad Bagherian

**Affiliations:** Department of Emergency Medicine, Mashhad University of Medical Science, Mashhad 9137913316, Iran

## Abstract

Spontaneous pneumomediastinum is an unusual and benign condition in which air is present in mediastinum. A 20-year-old male patient presented to ED with complaint of hoarseness and odynophagia from the day before, after weightlifting. The patient was nonsmoker and denied history of other diseases. On physical examination he had no dyspnea with normal vital signs. Throat examination and pulmonary auscultation were normal and no crepitation was palpable. We could not find subcutaneous emphysema in neck and chest examination. In neck and chest X-ray we found that air is present around the trachea. There was no apparent pneumothorax in CXR. In cervical and chest CT free air was present around trachea and in mediastinum. Subcutaneous emphysema was also evident. But there was no pneumothorax. The patient was admitted and went under close observation, oxygen therapy, and analgesic. The pneumomediastinum and subcutaneous emphysema gradually resolved within a week by conservative therapy and he was discharged without any complication. Many different conditions could be trigged because of pneumomediastinum but it is rarely seen in intense physical exertion such as weightlifting and bodybuilding. Two most common symptoms are retrosternal chest pain and dyspnea. But the patient here complained of hoarseness and odynophagia.

## 1. Introduction

Pneumomediastinum is a condition in which air is present in the mediastinum [1]. Its incidence is approximately 1 in 30,000 emergency department referrals [2]. Spontaneous pneumomediastinum (SPM) usually occurs with no underlying diseases or precipitating factors. It occurs more in young adults [3] with male to female ratio of 8 : 1 [4]. SPM associated with subcutaneous emphysema is rare and often benign [5]. Its diagnosis is very important because it may be associated with mediastinal organ injury [6]. SPM showed a possibility to convert to tension or malignant PM which may lead to cardiac or great vessel compression. SPM may be misdiagnosed because the most common presenting symptoms, chest pain and dyspnea, are signs of several of cardiopulmonary pathologies [7].

## 2. Case Presentation

**Figure 1:**
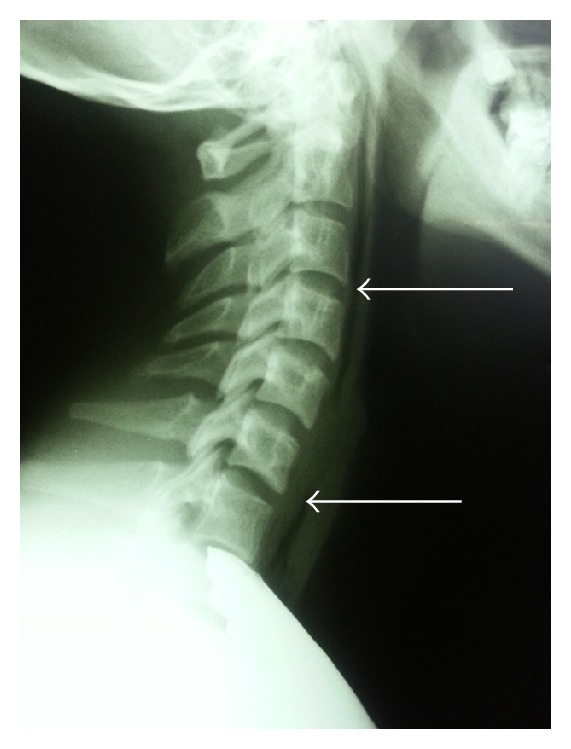
Neck X-ray, lateral view; free air is indicated by arrows.

**Figure 2:**
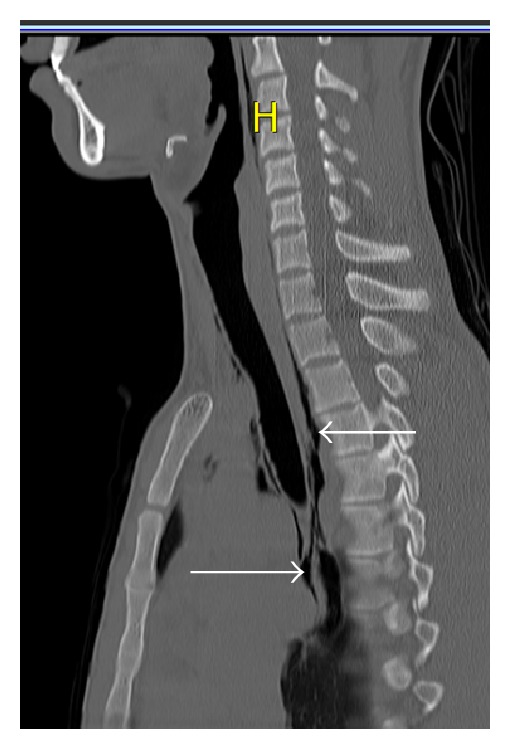
Neck and upper mediastinal CT scan, sagittal section; free air is present around trachea and in mediastinum (arrows).

**Figure 3:**
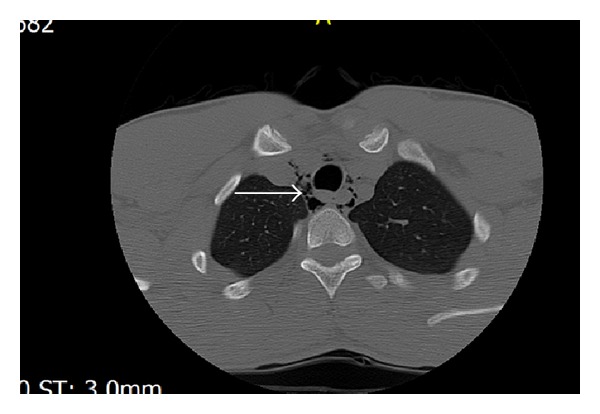
Mediastinal CT scan, axial section; free air is present around trachea and in mediastinum (arrow).

A 20-year-old male patient presented to ED with complaint of hoarseness, odynophagia, and neck pain from the day before, after weightlifting. The pain had begun about 6-7 hours after bench press. He had no extra lifting weights and everything was as normal as his every day practice. He applied to an ED center and was discharged with no important diagnosis. Due to progressive neck pain and dysphonia, he applied to this ED center. He reported pain while swallowing saliva or food. The patient was nonsmoker and denied history of other diseases. He received no medications and did not use illicit drugs. On physical examination he had no dyspnea with respiratory rate of 18 breaths per minute and was normotensive. Throat examination did not show any abnormality. Uvula and tonsils were normal without erythema. No lymphadenopathy or subcutaneous emphysema was palpable in neck examination. Pulmonary auscultation was normal with no decrease in breath sounds and no crepitation was palpable. Chest X-ray PA was done; there was no apparent pneumothorax. In neck X-ray (Figure 1), it is evident that air is present around the trachea. Chest CT was taken due to suspicion of pneumothorax. As seen, free air is present around the trachea and in mediastinum in sagittal and axial sections (Figures 2 and 3). Subcutaneous emphysema is also evident. But there is no pneumothorax. The patient went under conservative therapy with analgesic, rest, and oxygen. The pneumomediastinum was resolved within a week and the patient was discharged without any complication.

## 3. Discussion

The etiology of spontaneous pneumomediastinum is not well defined yet. SPM usually has a benign and favorable clinical course [8]. The trigger of pneumomediastinum could be asthma attack, barotrauma, intrathoracic pressure increase, the valsalva manoeuvre, and withdrawal symptoms of illicit drugs [9]. In this case it also happened after weight lifting which is associated with intrathoracic pressure increase. Overexpansion of distal airways and alveoli lead to alveolar rupture and the gradient between intraalveolar and perivascular interstitial pressure is the beginning of process of creating pneumomediastinum. Continuity of fascia through cervical soft tissue and mediastinum let the air spread through the neck and mediastinum during respiration and make the subcutaneous emphysema [10]. Two most common symptoms are retrosternal chest pain and dyspnea. But the patient here complained of hoarseness and odynophagia instead of chest pain or dyspnea. CT scan is considered a gold standard method for diagnosis of SPM [11]. The presence of air is obvious around the trachea and in mediastinum and neck in CT scan of the patient. The treatment consists of rest, analgesics, and close observation. The use of antibiotics, oxygen therapy, and dietary restriction is controversial [9]. The pneumomediastinum and subcutaneous emphysema gradually resolved within a week by conservative therapy and close observation.

Pneumomediastinum is a rare condition in young adults with unusual presenting symptoms. In young adults who apply to ED with any upper or lower respiratory complaint which could detect a trigger for SPM, SPM should be considered.
